# Supplementary material for: Are carbon emissions trading and green financial instruments synergistic? -Comprehensive quantitative research based on content analysis
Source: PLoS One. 2024 Mar 7;19(3):e0298601. doi: 10.1371/journal.pone.0298601 (PMC10919601; doi:10.1371/journal.pone.0298601)
Supplement: S1 Data — (ZIP) [file pone.0298601.s001.zip › Data/地级市Z_F工作报告.zip!/║┌┴·╜¡/╝╤─╛╦╣╩╨/└┤╫╘╣1⁄2╓┌║┼í╛┬φ┐╦╩2╛▌═°í┐_2019.docx]

各位代表：

现在，我代表市人民政府向大会报告工作，请予审议，并请各位市政协委员提出意见。

一、2018年工作回顾

过去一年，我们深入学习贯彻习近平新时代中国特色社会主义思想，认真落实党中央国务院、省委省政府和市委的要求部署，全力推动“农业富民、工业强市、城市提档、开放兴市”四大战略，统筹推进各项工作，全市经济社会保持平稳发展。2018年，预计全市地区生产总值同比增长4%，突破1000亿元大关；固定资产投资同比增长3.5%；一般公共预算收入同比增长3.8%；社会消费品零售总额同比增长8.1%；城乡居民人均可支配收入同比分别增长6.8%和7.2%；城镇登记失业率控制在3.95%。

——“三保”目标顺利实现。面对严峻的经济下行压力，特别是政府举债紧缩期、偿债高峰期、刚性支出快速增长期“三期叠加”的不利局面，把保工资、保运转、保民生作为压倒一切的任务，牢牢把握“抓收入、严支出、惠民生、保全局”总体要求，强力落实系列增收节支措施，保证了工资按时足额发放、各项运转经费支付和民生工程基本资金需求，有力防范了政府债务风险。强化经济运行调度，千方百计稳增长、促投资、强消费，经济总体平稳、稳中有进、进中向好。

——现代农业持续发展。农作物良种覆盖率保持98%以上，总投资26亿元的富锦锦西灌区年度建设任务顺利完成，粮食总产实现“十五连丰”，产能得到进一步提高。国家级绿色食品原料标准化生产基地面积达到890万亩，绿色食品标志总数达到270个，国家地理标志达到14个，“佳木斯大米”跻身2018中国区域品牌百强，农产品品质得到进一步提升。大力推进“农头工尾”“粮头食尾”，富锦和桦南30万吨燃料乙醇等粮食深加工项目落地，北京三聚环保等19家秸秆综合利用企业开工。全面启动农村人居环境整治三年行动，推进农村生活垃圾、厕所粪污治理，村容村貌得到显著提升。

——工业经济活力增强。在盘活存量上下功夫，打出“抓大、扶小、解困”组合拳，佳电股份、东兴煤化工税收分别超亿元，营业收入超亿元企业达到48户，新入规上和重返规上企业30户，降低企业生产经营成本3.29亿元。在扩大增量上做文章，山东汇盟新型除草剂、易郎汽车零部件等项目加快建设，中建材碲化镉弱光发电玻璃、太阳能基板玻璃等项目签约落地，全市开复工亿元以上产业项目111个，战略性新兴产业增加值同比增长11.6%。在创新驱动上求突破，实施高新技术企业双倍增三年培育计划，新增高新技术企业17户，通过国家科技型中小企业评价90户，新增省级科技创新基地12个。在园区建设上下力气，创新高新区管理体制和运营机制，国家级高新区创建取得积极进展，各县(市)区园区承载功能进一步增强。

——区域中心城市辐射功能有效提升。交通中心地位日益彰显。哈佳快速铁路建成通车，牡佳客专建设顺利推进，佳鹤铁路改造工程开工，佳木斯机场旅客吞吐量突破86万人次大关，创造历史新高，佳木斯站南北广场、佳西客运站投入使用。完成了和平街下穿、通江街通园巷公铁立交桥工程，有效缓解了市区南北通行压力。商贸物流中心作用初步显现。佳铁物流基地、京东支线智能云仓物流等项目进展顺利，创建电子商务产业园区7个、电商平台27个。健康医养中心供给能力增强。妇幼保健院、儿童医院新址正式启用，万里云医学影像诊断中心、宏大医院投入运营，建成医联体61个。科技创新中心初具规模。启动佳木斯工业技术研究院建设，培育佳木斯大学科技企业孵化器等一批创新平台，“双创”活力进一步释放。文化旅游中心日益活跃。舞剧《乌苏里传歌》在全国巡演32场，话剧《在路上》等一批文艺精品不断推出，快乐舞步健身操编创十周年邀请赛、海峡两岸校园足球邀请赛等赛事成功举办，中俄边境文化体育交流蓬勃开展。华夏东极被列为我省五大夏季精品线路，全年接待海外和国内游客收入分别增长3%和17.6%。教育中心实力凸显。落实教育优先发展战略，普惠性幼儿园覆盖率达到77%，义务教育均衡发展顺利通过国家验收，职业教育影响力增强，高考再创佳绩。

——开放合作步伐加快。中俄同江铁路大桥中方段主体工程全部完成，铁路口岸和港口东部正式作业区已完成国家相关部委会签，黑瞎子岛保护与开放发展总体规划获批。成功举办京·佳高端产业合作对接等系列活动，与中国科学院、广东省中山市、省农垦系统、哈尔滨工业大学、哈尔滨电气集团等合作共建全面推进，“俄罗斯企业佳木斯行”成效显著，与俄日等国家的合作交流范围持续拓展。新增外经贸主体69户。

——重点领域改革不断推进。全面落实政府承担的各项改革任务，谋划推动自主改革18项。深入推进审批服务便民化，公布“马上办、网上办、就近办、一次办”事项清单1035项，覆盖率98.3%，“一网通办”网上政务服务平台开通运行，“放管服”改革取得实效。市级机构改革扎实推进，市政府工作部门精简到34个，机构职能调整和人员转隶陆续到位，县(市)区机构改革全面启动。政府配置资源方式、桦南县相对集中行政许可权试点、乡镇政府服务能力建设等改革取得突破，政务服务进一步向基层延伸。

——群众获得感切实增强。贫困人口稳定脱贫。当年实现18367名贫困人口精准脱贫，贫困发生率降至0.69%。抚远市顺利脱贫摘帽，同江、桦南、汤原、桦川四个国贫县完成市级初审核查。民生实事有效完成。在财政压力不断加大的情况下，优先民生投入，承诺的民生实事得到高效落实，超额完成预期计划。全力推进暖屋子工程。围绕保供、保热、保满意，改造老旧管网57公里，整改温度不达标楼房136万平方米，投产永旭热源，加强行业管理，实行供热质量考核，信访投诉较上个供热期大幅下降。市民出行更加方便。和平街北段道路及雨水泵站工程完工，圃东街南段主干道路通车，维修街道50余条，新增电动公交车170台，施划停车位1236个。人居条件显著改善。集中改造棚户区9569套、农村危(草)房3968户、老旧小区124栋66万平方米、农村居民室内厕所8640个，住房公积金贡献率118.48%。生态环境日益优美。开展减霾卫蓝、治污净水、保土护田三大行动，实行最严格的秸秆禁烧制度，全市环境空气优良天数较去年增加13天，松花江水污染防治成效明显，国控重点污染源废水排放达标率100%，建成区绿化覆盖率达到41.2%。积极解决群众关心的问题。已回迁安置居民3291户，办理房屋产权证15950户，房地产领域信访问题专项整治取得新成效。公立医院全部取消药品加成。全面整合城乡居民医疗保险和大病保险。果断处置炭疽病、非洲猪瘟疫情。社会治安案件和刑事案件、受理群众信访和越级非访双双大幅下降，扫黑除恶专项斗争取得阶段性战果，社会保持和谐稳定。

过去一年，我们坚持党的全面领导，坚决在思想上政治上行动上同以习近平同志为核心的党中央保持高度一致。自觉接受人大和政协监督，定期向人大及其常委会报告工作，向人民政协通报情况，市人大代表建议、市政协委员提案办结率100%。建立市政府常务会会前学法制度，完善重大决策、合法性审查的程序和机制，全面完成现行规范性文件的清理工作，依法行政水平进一步提高。认真落实全面从严治党要求，切实推进党风廉政建设，大力优化营商环境，深入开展机关作风整顿，政府职能和作风进一步转变。

国防动员和后备力量建设、双拥工作、司法行政、统计监测、外事侨务、民族宗教、档案史志、防灾减灾、地震气象、人民防空等各项工作，以及妇女儿童、老龄和残疾人等事业都取得了新的进展。

各位代表！回顾过去一年，我们顶住了压力，挺过了艰难，成绩来之不易。这是坚定以习近平新时代中国特色社会主义思想武装头脑、指导实践、推动工作的结果，是省委省政府和市委正确领导、科学决策的结果，是全市人民团结奋斗、开拓进取的结果；是市人大法律监督、工作监督和市政协民主监督、参政议政的结果；是驻佳部队、中省直单位、各民主党派、人民团体、社会各界人士、外来投资者和建设者关心支持的结果。在此，我代表市政府，向为佳木斯振兴发展作出贡献的全市人民和各界人士表示衷心感谢！

同时，我们也清醒地认识到，当前，佳木斯的振兴发展正处在爬坡过坎的关键时期，保持经济社会持续健康发展的基础还不稳固，受经济下行形势影响，去年地区生产总值、固定资产投资等经济指标没有完成全年预期目标，给今年工作带来更大的压力。经济总量偏小、经济结构偏重、经济实力偏弱，将是我们相当一段时间要面对的突出问题；产业项目储备不足、投资需求拉动不足、创新要素支撑不足，没有真正形成新的增长极；县域经济比较薄弱、开放型经济比较薄弱、民营经济比较薄弱，一些企业生产经营困难；基础设施欠帐较多、民生保障欠帐较多、生态治理欠帐较多，群众还有不少不满意的地方；有的部门职能转变不到位，优化营商环境、提高行政服务水平等工作亟待加强和完善。我们必须直面这些问题，知难而进，迎难而上，采取有力措施加以解决。

二、2019年工作安排

2019年是新中国成立70周年，是全面建成小康社会关键之年。既要看到，外部环境复杂多变，内部新老问题交织，经济下行压力加大，佳木斯发展仍面临着严峻挑战；更要看到，我们正处于重大机遇集中期。习近平总书记在深入推进东北振兴座谈会上提出的新要求和近期召开的中央经济工作会议，为东北全面振兴全方位振兴提供了新的政策机遇，大规模的投资需求将对新一轮经济增长发挥关键作用，振兴发展的外部助推力正在增强；全市经济在转型阵痛中优化升级，相继落地的一批重大项目将起到较强的拉动作用，积极创建的国家级高新区将成为放大产业集群效应的重要载体，全力打造的“一桥一岛”开辟了对外开放的新窗口，振兴发展的内部支撑力正在增强；哈佳快速铁路顺利运营、牡佳客专加快建设，必将带来我市与省会城市圈“同城效应”的发酵、与省内各大经济板块的互通共融，促进人流、物流、资金流加速向我市汇集，推动项目、技术、人才不断向我市涌动，振兴发展的区域影响力正在增强。我们既要增强忧患意识，善于化危为机，又要坚定信心，抓住机遇，全力把佳木斯的事情办好。

2019年工作总的要求是：坚持以习近平新时代中国特色社会主义思想和党的十九大精神为指导，全面贯彻落实习近平总书记在深入推进东北振兴座谈会上的重要讲话和考察黑龙江的重要指示精神，全面落实中央经济工作会议精神和省委省政府各项决策部署，全面落实市委十三届五次全会精神，认真践行新发展理念和高质量发展要求，坚持稳中求进工作总基调，以供给侧结构性改革为主线，坚持市场化改革，扩大高水平开放，深入解放思想，提振市场信心，在推进落实“四大战略”实践中，努力开拓农业富民新路径，培育工业强市新动能，强化城市提档新支撑，构建开放兴市新格局，全力推进佳木斯全面振兴全方位振兴，以优异成绩庆祝新中国成立70周年。

综合考虑各方面实际情况，今年全市主要预期目标是：地区生产总值增长5.5%左右；居民消费价格涨幅控制在3%左右；城镇新增就业3.5万人，城镇登记失业率4%以内；城乡居民可支配收入增长高于经济增速；单位地区生产总值能耗下降3%左右。

(一)加快把农业优势转化为经济优势，开拓农业富民新路径。坚持把实施乡村振兴战略作为新时代“三农”工作总抓手，推进质量兴农、绿色兴农、品牌强农，促进农业全面升级、农村全面进步、农民全面发展。

以提高农产品质量为主攻方向，走绿色化、优质化、特色化、品牌化发展的路子。着眼提高农业比较效益，落实“三减”面积380万亩，绿色食品基地面积发展到900万亩。围绕发展现代农业建设高标准农田，提高基础设施保障能力，加快锦西、临江、三村、乌苏镇灌区及田间配套工程建设，压减地下水用量2亿立方米，粮食综合生产能力稳定在140亿斤。依靠农业科研力量，加强重点良种培育、关键农机研发、特色产品开发，提高成果转化率，完成佳木斯国家农业科技园区验收，打造质量兴农引领平台。面向中高端市场，加强农产品质量安全监管，提高优质专用粮食比重，发展绿色生态健康养殖，强化品牌培育塑造，打响“佳木斯大米”“佳木斯大豆”“佳木斯木耳”等特色区域品牌，助推好产品卖出好价钱。毫不放松粮食生产，落实粮食安全行政首长负责制，为我省当好国家粮食“压舱石”做出贡献。

以农业全产业链增值为着眼点，做好一二三产业融合发展大文章。深入贯彻习近平总书记考察我省的重要讲话和重要指示精神，全力推进“农头工尾”“粮头食尾”，通过大力发展粮食加工业、农产品精深加工、新型流通业态，延伸产业链、打造供应链、提高附加值。大力推进玉米向燃料乙醇、生物制药等产业链条下游延伸，成立市代餐粉产业联盟，带动代餐粉产业做大做强，推动建设非转基因大豆深加工产业基地，推行种养加一体化精深加工，力争全市农产品加工业实现主营业务收入200亿元。加大秸秆“五化”综合利用力度。积极发展连锁经营、专营门店、农村电商等流通方式和流通业态，实现产销对接。深入挖掘农业的多功能性，创新发展休闲农业、乡村旅游、生态康养等新业态，大力培育特色消费、共享经济、体验服务等新增长点，不断拓宽农民增收渠道。

以健全农业社会化服务体系为抓手，着力培育壮大新型农业经营主体。充分发挥农业技术推广体系作用，鼓励各类组织开展农机作业、集中育秧、生产托管、加工储存等服务，推动农业生产性服务业发展。积极发展农民合作社、家庭农场、种养大户，支持县乡村大胆创造适度规模经营的实现形式，创建“一乡一业、一村一品”特色优势产区，在专、精、特上做文章，带动提高产业整体规模和区域整体效益。推进农村集体产权改革，创新农村集体经济运行机制，增加农民财产性收入。

以打好脱贫攻坚战为第一任务，实现精准退出和脱贫后稳固提升。坚持目标不变，靶心不散，频道不换，坚决打赢脱贫攻坚战，确保今年顺利通过国省检查评估，实现4个国贫县全部摘帽。进一步强化产业扶持，引导扶贫资金和涉农整合资金向产业项目倾斜，创新带贫益贫机制，保证贫困人口实现长期稳定脱贫。加大对有劳动能力和就业意愿贫困户的职业技能培训力度，建立长效帮扶机制，防止返贫和产生新的贫困。逐步把针对绝对贫困的脱贫攻坚过渡到针对相对贫困的日常帮扶措施，纳入乡村振兴战略框架内统筹衔接，选派驻村产业指导员，提高贫困乡村、贫困人口自我发展能力。

以实施乡村振兴战略为统领，大力改善农民生产生活条件。统筹落实乡村振兴战略，科学规划，分步实施，补齐短板。深入推进农村人居环境整治三年行动，加快改变“脏乱差”的面貌，努力创建干净整洁、美丽宜居的农村生活环境。抓好垃圾集中处理，梯次推进农村生活污水治理，健全厕所粪污收集处理机制，探索市场化运作模式。毫不松懈抓好非洲猪瘟防控，加强畜禽养殖废弃物资源化利用，引导农户退散养、退庭院，进规模场、进合作社，促进绿色养殖发展。完成绿化村庄350个，示范村绿化覆盖率达到20%，达标村达到15%。

(二)加快把工业发展要素转化为经济优势，培育工业强市新动能。积极引进战略投资者，强化企业创新主体地位，激活水、电、土地、环境容量、人力资源等产业要素，全面做好“三篇大文章”，加快实现新旧动能转换，着力做大做强做优实体经济。

实施企业升规攻坚。把规上企业培育作为工业扩量提效的基础工作，在提升企业经营水平、扩大经营规模上攻坚。加强对临规企业、成长型企业、退规企业、漏统企业的跟踪监控及协调服务，组织开展多种形式合作对接、供需对接、配套对接，认真落实更大规模减税降费政策，支持困难企业扭亏增盈、中小企业发展壮大，推动一批企业小转初、初转规，力争新增规上企业30户。加紧国家火炬计划农机特色产业基地、佳木斯大学国家级科技园和孵化器创建，加快佳木斯工业技术研究院等科技创新平台建设，实施新一轮“科技型企业三年行动计划”和“高新技术企业双倍增三年培育计划”，精准扶持一批科技型、创新型企业。推进电机股份、象屿金谷、恒源食品、百威英博、佳星玻璃等企业改扩建项目，带动区域传统产业“二次创业”、转型升级。加快润特科技水溶性铝、葵花药业原料药生产基地等项目建设，力争战略性新兴产业增加值增长15%。加快处置“僵尸企业”，推进龙江福浆纸、乌苏里江制药、赛瑞南华糖业等闲置资产盘活项目，让沉睡资源重现活力。

开展产业项目招商攻坚。把产业项目招商建设作为经济工作首要目标和任务，围绕新一轮产业项目三年行动计划，在产业项目招商攻坚中取得突破。谋划引进一批农副产品精深加工、现代装备制造、清洁能源、新材料、生物医药等项目，形成“实施一批、建成一批、储备一批”的梯次推进格局，力争全年开复工亿元以上项目140个以上。实施专业招商，组建专业招商队伍，实行“专班+园区”“目标+考核”机制，制定市场化的绩效激励政策。加强产业链招商，细分重点产业，找准与发达地区产业转移的承接点，进行有针对性的小分队、敲门式招商。推行以商招商，围绕龙头企业开展招商，激发产业联盟、企业协会等组织活力，推动产业协同发展，形成产业集聚效应，做大产业集群。

打好工业立县攻坚。把“工业立县”作为加快县域经济发展的重中之重，形成全力攻坚的良好氛围。坚持一手抓扶优扶强，一手抓优化整合，每个县(市)区重点培育2个以上立县主导产业，完善扶持民营企业发展的具体政策，培育企业家队伍，补齐县域工业经济偏弱的短板。坚持一手抓导向，一手抓机制，通过政府引导和市场主导，促进各县(市)区发展特色产业基地，建立健全产业项目信息上下联动机制，确保大项目、好项目留得住、落得下。进一步加大对各县(市)区新增规上企业户数、工业经济指标增幅的考核权重，强化争先意识，选准省内县份的参照系，力争全省排名晋位，形成对全市经济的正向拉动。

加快园区升级攻坚。把园区经济作为工业重要增长极，全力推动佳木斯国家级高新区以升促建。引入济南高新区成熟经验，健全体制机制，完善综合功能，真正把高新区打造成产业集聚、企业汇聚、人才广聚的创新转型高地。加强与哈尔滨工业大学、省工业技术研究院、哈尔滨电气集团合作，推动科技成果转化。实行全员招商，全力促成项目落地，力争年内实现10个项目建成投产、20个项目开工建设，完成固定资产投资21亿元、技工贸总收入190亿元、地区生产总值45亿元。按照特色化、专业化、集约化发展方向，引导各县(市)区因地制宜、注重质量，加强园区优化整合、扩容升级，培育发展拉动作用强、市场潜力大的好项目、好企业。

(三)加快把区位优势转化为经济优势，强化城市提档新支撑。突出区域中心城市定位，围绕发挥“六个中心”功能，努力打造宜居、宜业、宜商、宜游、宜养的美丽生态城市。

强化城乡规划引领。以提升城市辐射功能、建设优良人居环境为中心，高起点做好国土空间规划的谋划与编制工作，统筹优化各类空间规划布局，推进城乡整合、多规合一，做好新一轮城市总体规划编修工作，增强规划的科学性、前瞻性、约束性。合理安排年度新增建设用地指标，满足经济建设需求，预留城市发展空间。

强化基础设施建设。抢抓国家加大基础设施投入的窗口期，着力加强重点基础设施项目建设。加快推进公铁联运客运枢纽、国省道改扩建、松花江二桥等项目，抓好佳木斯机场建设和新增航线航班工作。优先建设城市道路交通、城市管网等与民生密切相关的基础设施，提高城市综合承载能力。放宽市场准入门槛，鼓励社会资本参与PPP项目，推动政府主导下的多元经营主体的资本化市场运作，促进“以城建城、以城养城”的良性循环。

强化城市管理。城市让生活更美好，管理让城市更美丽。加强交通管理，治理重点路段，打通“主动脉”，改善“微循环”，让交通“畅”起来。坚持晨清扫、日保洁，整治背街小巷卫生，推进餐厨垃圾处理，强化绿色清雪，让城市“净”起来。抓好“一轴三带、四区、多点”亮化改造，合理搭配灯品灯饰，让城市“靓”起来。对林海路等8条新建街路、长安路等27条主要街路进行绿化和补植，让城市“绿”起来。整治乱贴乱画、乱搭乱建、乱停乱靠，让城市秩序“好”起来。加强房地产市场管理，严格市场准入机制，强化商品房预售资金监管，规范房地产市场秩序。加强物业管理，继续推进棚户区改造，做好公租房工作，用好用足公积金，不断完善住房保障体系。

强化辐射服务功能。以提升城市承载力、辐射力、影响力为目标，引领现代服务业发展。规划建设高铁南站新商圈，加快铁路物流基地项目建设，带动物流业不断繁荣。推动多层次资本市场建设，努力扩大直接融资规模。深化会展营销、信息科技、文化创意等领域创新创业，带动新产业不断兴起。推进普惠性学前教育和义务教育优质均衡发展，构建现代化职业教育体系；加强基层医疗机构标准化建设，推进公立医院改革，建立现代医院管理制度；统筹推进新时代文明实践中心、三江文化展示中心、基层综合文化服务中心建设；深入实施全民健身计划，打造“佳木斯快乐舞步”城市名片，积极参加和举办各类国内外赛事，带动公共服务事业全面上水平。

(四)加快把口岸优势转化为经济优势，构建开放兴市新格局。积极融入国家“一带一路”“中蒙俄经济走廊”和全省“打造一个窗口、建设四个区”，全力推动“一桥一岛”核心区落成和辐射半径延伸，全方位促进以对俄合作为重点的大开放。

着力建设大桥经济区。抓住中俄同江铁路大桥开通带来的对俄开放新机遇，举全市之力，加快推进跨境合作区和桥头经济区建设。扩展大桥经济区的内涵和外延，在空间上形成沿松花江、黑龙江对外开放经济带，构建佳木斯市区、汤原、桦南、桦川、富锦、同江、抚远为一体的大桥经济新格局，实现资源共享、优势互补、协同发展；在内涵上丰富对外开放新内容，按照“原字号”进口、进口抓落地，“工字头”出口、出口抓加工的思路，超前做好货源组织，切实满足大桥通车运营需求。积极创建国家级边境经济合作区、综合保税区和跨境经济合作区，深化与国储能源、中林集团等大企业合作，整体开发农副产品加工产业园、木材加工制造产业园、能源及化工产业园、国际物流产业园、进出口产品综合加工产业园，努力形成从贸易到加工再到产业的全产业链发展。各县(市)区要主动融入大桥经济时代，不问所在所有，但求所用所为，切实参与大桥经济区建设，借助国际大通道，促进全域大开放。

着力推进黑瞎子岛保护开发。始终把黑瞎子岛生态环境保护摆在首位，进一步加强生态修复、水系疏通、动植物保护等工作，保持好原始风貌。坚持市场化运作、公司化运营，推进黑瞎子岛防洪排水工程和配套设施建设。加快黑瞎子岛陆路口岸相关手续审批，力争陆路客运口岸早日开通。高起点、特色化建设黑瞎子岛镇，谋划推动建设国际旅游岛，以人流带动物流、资金流。深入实施兴边富民行动，抢抓国家政策倾斜的机遇，积极争取边境基础设施建设投资，集聚边境地区人口和生产要素，发展特色优势产业，提升沿边开发开放水平。

着力深化多层次合作交流。继续深化垦地合作，加快“大三江一体化”进程。融入哈尔滨“两小时”经济圈，带动我省东部城市群，强化功能互补、要素共享、产业关联。进一步推动与广东省中山市对口合作项目的具体落实，力争在共建高新技术产业园、建设“飞地经济”和重大项目合作等方面取得实质性进展，在面向粤港澳大湾区的合作交流上取得突破。加强与日韩、欧美等国家的经济合作和人文交流，促进对外开放向更宽领域、更高层次拓展。

(五)加快把生态优势转化为经济优势，践行绿色发展新理念。深刻理解习近平总书记关于“绿水青山就是金山银山，冰天雪地也是金山银山”理念的丰富内涵，把生态文明建设融入到经济社会发展全过程，切实保护好、充分利用好我市宝贵的原生态资源。

优化生态环境质量。聚焦中央环保督察“回头看”反馈意见整改，开展生态环境整治提升年活动。打好蓝天保卫战，强化燃煤治理和燃煤锅炉淘汰改造，严格建筑扬尘管控，持续推进秸秆露天禁烧，实现空气更清新。打好碧水保卫战，全面落实河湖长制，实施“一河(湖)一策”治理方案，推进松花江流域污染治理和污染减排项目建设，强化城乡污水、黑臭水体治理，实现河水更清澈。打好净土保卫战，实施黑土耕地保护三年行动计划，抓好土壤污染状况详查、土壤环境管理，加强土壤污染治理与修复，实现黑土更清洁。深入推进“大棚房”问题清理整治，坚守农地姓农底线。

加快发展全域旅游。把发展全域旅游作为实现全市资源整合、城乡融合、促进经济社会协调发展的重要举措加以推进。做好全域旅游规划。委托高水平专业机构开展全域旅游规划设计，统筹“吃住行游购娱”、“商养学闲情奇”，开发红色文化游、森林游、边境游、湿地游、康养游、冰雪游、民俗游等旅游产品，精心打造“华夏东极”旅游品牌，切实解决旅游要素融合不够、旅游线路细碎化等问题。培育旅游产业主体。组建文旅集团，引进战略投资者，发展壮大旅行社，开展专题推介活动，发挥“文旅+”融合作用，加快新产能集聚。强化旅游市场监管。建立健全权责明确、执法有力、行为规范、保障有效的旅游市场监管机制，大力营造诚信经营、公平竞争、文明有序的旅游市场环境，不断扩大佳木斯旅游品牌影响力。

(六)加大营商环境建设力度，努力实现向好拐点转变，夯实振兴发展新保障。深入实施“四大战略”，把市委确定的宏伟蓝图变成美好现实，必须全面正确履行政府职能，统筹改革发展稳定各项工作，努力使软件硬件、综合实力全面强起来，为高质量发展提供坚实的保障。

竭力优化营商环境。坚持向营商环境要竞争力，争取营商环境向好变化的拐点在佳木斯早日出现。坚持深化“放管服”改革，以“放”为原则，以“不放”为特例，持续简政放权，推动审批服务便民化，实现多数事项“网上办”，必要事项窗口“一次办”，少数企业特殊审批事项“我帮办”。推行多证合一、证照分离，推进市场主体登记全程电子化。扩大“双随机、一公开”监督方式覆盖面，加强事中事后监管，对符合市场准入条件的所有企业，只开绿灯、不设路障，有求必应、无事不扰，靠前服务、关系亲清。坚持开展营商环境评价，加大营商环境受理举报事项督办力度，严厉惩处各类破坏发展环境的行为。完善守信激励与失信惩戒机制，对失信企业实行信用“黑名单”制，建设诚信佳木斯。

全面深化重点改革。坚持向改革创新要发展，积极解决不适应市场经济要求的体制机制等深层次矛盾和问题。认真抓好中央和省各项改革政策的承接落实。深入推进投融资体制、财税金融体制、国有资本管理运营体制、农村土地制度等改革，创新引资引才机制。积极发展混合所有制、股份合作制经济，加大对中小企业创新支持力度，激发微观经济主体活力。继续深化社会体制、生态文明体制等领域改革，加强和改进退役军人事务工作，把各方面的发展潜力充分释放出来。进一步向基层放权，尊重基层和群众首创精神，激发基层改革创新活力。

倾心办好民生实事。坚持保障和改善民生，不断提高人民群众的获得感、幸福感、安全感。重点办好10个方面民生实事：①打通和平街、北纺街等南北通道，拓宽改造育林路、新华街，疏通胜利路环岛等低洼路段排水系统，治理城市交通拥堵，解决群众出行难。②实现国家级食品安全示范城创建目标，完成江北新水源地项目建设，保障群众饮食安全。③新增公租房租赁补贴1000户，改造农村危房2300户以上，完成农村居民室内改厕7000户以上，改造老旧小区楼房300栋，对100栋“弃管楼”实施市场化物业管理，改善城乡居民居住条件。④推行燃气用户保险，推进报警器安装，改造和新建燃气管网，强化燃气安全管理。⑤新增城镇就业3.5万人，扶持大学生创业，增加创业小额担保贷款投放，加大就业创业服务力度。⑥继续实施暖屋子工程，严格供热企业考核监管，改造老旧供热管网，将居民室内供热标准提高到20℃。⑦强化基层卫生医疗机构功能，充实社区卫生服务中心医疗人员50人，加快推进家庭医生签约服务，建设2所标准化县级妇幼保健机构、2所二级甲等中医院，方便群众就近就医。⑧继续实施阳光分班，切实减轻中小学生课业负担，完成第一中学新教学楼建设，优化教育资源配置。⑨新建1000平方米省级城市示范社区1个，新建改建500平方米省级农村样板社区9个，打造社区“15分钟生活服务圈”，拓宽社区服务领域。⑩全市建设“爱心驿站”100个，解决环卫工人、快递员等户外工作者如厕休息难问题，体现人文关怀。

切实加强自身建设。努力建设人民满意的法治政府、创新政府、廉洁政府和服务型政府。持续深入贯彻落实习近平总书记重要讲话和重要指示精神，牢固树立“四个意识”，坚定“四个自信”、坚决做到“两个维护”。坚决贯彻落实市委决策部署，自觉做到不越位、不错位、不空位，撸起袖子干到位。主动接受人大的法律监督、工作监督和政协的民主监督，认真办理人大代表建议和政协委员提案，充分听取各界各方意见。坚持问需于民、问政于民，加大重点领域政务信息公开力度，推进严格规范公正文明执法。认真开展以解放思想推动高质量发展为主题的“大学习、大讨论、大调研”活动，加强政府系统党风廉政建设和反腐败斗争，激励干部担当作为，确保各项任务落地见效。

各位代表，新时代要有新气象，新起点要有新作为。让我们更加紧密地团结在以习近平同志为核心的党中央周围，在省委省政府和市委的正确领导下，解放思想，改革创新，汇聚起全市人民的磅礴力量，奋力开创佳木斯全面振兴全方位振兴的崭新局面！
